# Supplementary material for: Protein Phosphatase OsPP2C55 Negatively Regulates Abscisic Acid Biosynthesis and Saline–Alkaline Tolerance in Rice
Source: Plants (Basel). 2025 Nov 3;14(21):3362. doi: 10.3390/plants14213362 (PMC12610132; doi:10.3390/plants14213362)
Supplement: Supplementary file 1 [file plants-14-03362-s001.zip › 10.30 Revised supplementary material.pdf]

Supplementary Material

1 Supplementary Figures

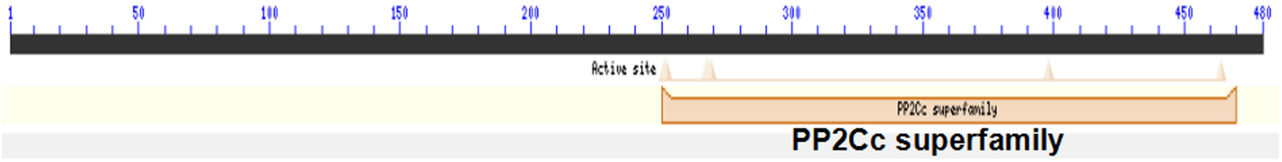

Supplementary Figure S1. Conserved domain of OsPP2C55 protein. The NCBI database was used (https://www.ncbi.nlm.nih.gov/Structure/cdd/wrpsb.cgi) to depict the conserved domains of OsPP2C55 protein.

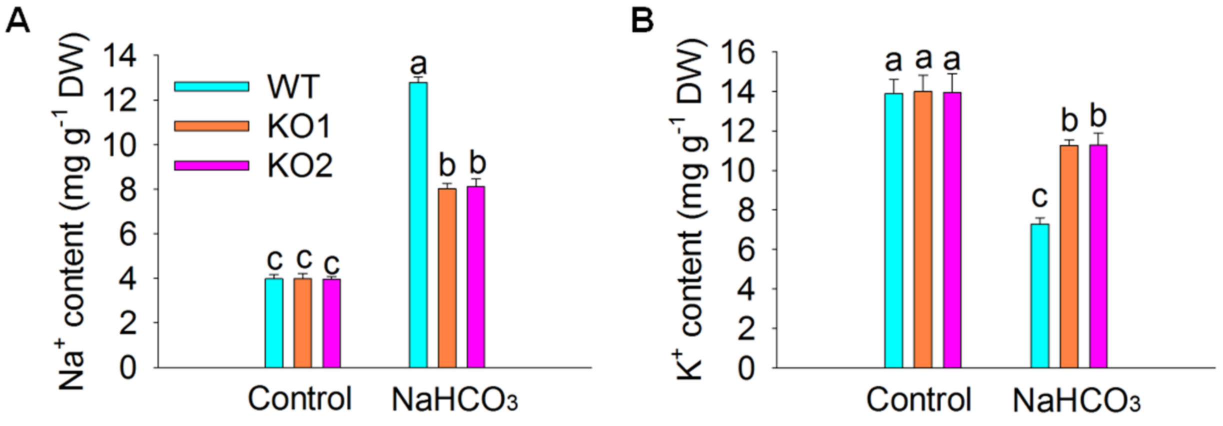

Supplementary Figure S2. Na^+ and K^+ content of *ospp2c55*-KO (KO1 and KO2) and WT plants under saline-alkaline stress. **(A, B)** The content of Na^+ (A) or K^+ (B) in the leaves of *ospp2c55*-KO (KO1 and KO2) and WT plants treated with 100 mM $NaHCO_3$ for 24 h. The content of Na^+ or K^+ was analyzed using an inductively coupled plasma-optical emission spectrometry instrument (ICP-OES; Optima 8000, Perkin Elmer, USA). The results of all experiments were repeated at least three times. Data in (A, B) are means ± SD of three replicates. Different letters indicate significant differences at the *$P<0.05$* level according to one-way ANOVA.

2 Supplementary Tables

Supplementary Table S1. Primers in this study.

Genes	Primers for	Sequence (5'-3')
OsABA2	pGBKT7	F:GAATTCATGTCCGCCGCCGCCGCCGCC
		R:GGATCC TTAATCTTCAAATGCTCTC
OsPP2C55	pGADT7	F:GAATTCATGCTTGCCGGCGGAGGAAG
		R: GGATCCTCATGTAGCTGAGGCGCTTGT
OsPP2C55	pCAMBIA1300-nLUC	F: GGATCCATGCTTGCCGGCGGAGGAAG
		R:GTCGACTGTAGCTGAGGCGCTTGTCAC
OsABA2	pCAMBIA1300-cLUC	F:GGTACCATGTCCGCCGCCGCCGCCGCC
		R:GGATCC TTAATCTTCAAATGCTCTC
OsPP2C55	qRT-PCR	F:TCCTGGCGACGTTATTATTG
		R:TGTTCCTATCCATTGCCTTTT
OsACTIN	qRT-PCR	F:CTTCATAGGAATGGAAGCTGCGGGTA
		R:CGACCACCTTGATCTTCATGCTGCTA
Ospp2c55	Identification of CRISPR-cas9	F:TCCCGAAATTGTTGCGGTCT
	knockout materials	R:TCTGTGGCTCCAGCAGAGTA
OsNCED1	qRT-PCR	F:TCGCCATCACCGAGAACTA
		R:TCTCCTGGAGCTTGAACACC
OsNCED3	qRT-PCR	F:CCCCTCCCAAACCATCCAAAC
		R:TGTGAGCATATCCTGGCGTCGT
OsABA2	qRT-PCR	F:TCATTCATACACAGCAACCAAGCAT
		R:CTTCACCACCAACAAAGGCGAAA
OsABA8ox2	qRT-PCR	F:GCGAGACGCTCCAGCTCT
		R:GGGCACCCCAGCAGATT
OsSODCc2	qRT-PCR	F:GGAGAAGATGGTGTTGCTA
		R:GCCTTGAAGTCCGATGAT
OsCatB	qRT-PCR	F:GGCTGTCGGGAAAAGTGTGTCATTG
		R:TTTCAGGTTGAGACGTGAAGCCAGC
